# Disseminating Health Prevention Programs Using AI-Crafted, Culturally Tailored Ads for Somali American Adolescents

**DOI:** 10.1007/s11121-026-01891-6

**Published:** 2026-03-18

**Authors:** Tori Simenec, Salma Ibrahim, Gail Ferguson

**Affiliations:** University of Minnesota, Minneapolis, MN USA

**Keywords:** Digital interventions, Dissemination, Cultural adaptation, Immigrant/refugee youth, Adolescents

## Abstract

Recent advancements in artificial intelligence (AI) and digital health messaging present opportunities to bridge service health gaps in underserved communities. Adolescents are highly engaged with technology; however, they lack the access to relevant health information and resources tailored to their specific needs. This may be especially true for Black migrant-background adolescents. Utilizing publicly available AI resources can support the dissemination of culturally responsive digital prevention programs. The present study developed four 1-min direct-to-consumer (DTC) video health advertisements that varied in cultural and developmental tailoring, using focus-group-generated digital health message preferences. The advertisements promoted a culturally adapted food-focused media literacy program, JUS Media? Global Classroom – Somali American. The four ads were experimentally tested among 230 Somali American adolescents (ages 11–19, M_age_ = 14.9; 67.5% female) who through age-stratified randomization viewed the DTC video ads. Surveys assessed personal relevance, elaboration (i.e., critical thinking after receiving the message), message liking, and intentions to use the advertised online health intervention. Hierarchical regression analyses revealed that cultural adaptation significantly increased three of four primary indicators of program effectiveness—personal relevance, liking, and usage intentions—whereas developmental adaptation did not significantly predict outcome variables. Insights from the study offer novel strategies in applying human-centered AI to co-develop tailored digital advertisements promoting digital prevention programs. Cultural tailoring of health messages has been criticized for requiring a substantial degree of human and financial capital. Publicly available AI tools can efficiently design tailored develop tailored direct-to-consumer (DTC) ads to disseminate digital health programs and overcome logistical barriers. This is important given delivering health interventions to youth, perhaps especially Black migrant-background youth, is often challenging due to institutional gatekeepers and lack of culturally responsive interventions and recruitment/dissemination strategies (Hill, L., Ndugga, N., & Artiga, S. (2024, June 11). Key data on health and health care by race and ethnicity. KFF. https://www.kff.org/key-data-on-health-and-health-care-by-race-and-ethnicity/). To propel accessibility and scalability of services to support Black migrant youth in the U.S., who are at risk for unhealthy eating due to disproportionate targeting of unhealthy food messages, prevention and intervention programs can capitalize on the very same tactics used by junk food advertisers to strategically develop and disseminate health services. By leveraging Artificial Intelligence (AI) to develop DTC marketing of digital prevention programs, service providers can effectively reach audiences most likely to benefit, offering a more cost-effective alternative to traditional recruitment strategies (Simenec et al., Journal of Child and Family Studies 32:1425–1437, 2023). Most research on tailored DTC strategies has been conducted with adults and only after intervention efficacy studies, delaying the reach/impact of targeted youth health programs. This experimental study investigates the effectiveness of culturally and developmentally tailored DTC video ads for a digital food-focused media literacy program for Somali American adolescents. Ads were developed with the support of AI tools and in partnership with the Somali American community.

**Supplementary Information:**

The online version contains supplementary material available at https://doi.org/10.1007/s11121-026-01891-6.

## Digital Health Interventions for Somali American Adolescents

Despite the United States investing billions of dollars in the early 2000 s to expand access to evidence-based programs (McHugh & Barlow, 2010), the demand for these services has far outpaced the availability of trained professionals, creating a persistent supply-and-demand dilemma. The U.S. healthcare system largely uses a one-size-fits-all model of delivery, which is ladened with barriers to care and interventions designed for White, well-resourced, individuals (Hill et al., 2024). Consequently, treatment gaps are most striking in racially marginalized populations, including migrant-background youth of color, who are less likely than their White peers to make contact with service providers (Wang et al., 2005). U.S. structural racism presents Black youth with many risks to their health, and while evidence-based resources designed to buffer these risks exist, barriers to access and engagement persist (Hill et al., 2024).

The use of digital platforms to deliver health services can overcome barriers to care by providing accessible, low- or no-cost services that are easily adapted to appropriate developmental and cultural contexts (Simenec et al., 2023). Black adolescents report finding digital health tools acceptable and desirable when seeking health services (Willis & Neblett, 2023). This is especially relevant in this current era since the use of online health resources and interventions has skyrocketed since the COVID-19 pandemic (Wijesooriya et al., 2020). Such resources have been shown to impact adolescent knowledge, attitudes, and intentions to protect their health in a variety of health behaviors (Choi et al., 2023; Ma et al., 2023).

The Somali American community is one group who may benefit from digital health resources. Following two significant waves of involuntary migration—first in 1960 after Somalia's independence and again in 1991 due to civil strife—Somali American youth represent both first-generation migrants, who have firsthand experience of migration-related adversities, and second-generation individuals born in the U.S., who confront post-migration issues such as racism and limited access to culturally relevant health resources (Abdi et al., 2022; Hodges et al., 2024). When in need of support, Somali American adolescents indicate seeking help primarily through informal relational supports (e.g., family, school, friends) or religion (e.g., religious leaders, prayer; Ellis et al., 2010). Common barriers to accessing formal health care include linguistic barriers, distrust of cultural outsiders, and not being included in the development of health services for their community (Abdi et al., 2022). However, growing up as digital natives, Somali American adolescents are likely exposed to a substantial amount of digital health messages given that a recent study found that Black migrant adolescents, including Somali American youth, spend 52.2 h per week using screen media (Ahn et al., 2023). This suggests that tailored DTC health messages may support health prevention efforts by bolstering recruitment, access, and uptake of digital interventions for Somali American adolescents.

### Message Tailoring

Message tailoring is defined as using any combination of characteristics about an individual to alter communication in a way that maximizes the effectiveness of the message in favor of targeted outcomes (Petty et al., 2009). This may include incorporating contextual and demographic factors like culture, age, gender, or personal characteristics into a message’s content. Importantly, a higher degree of tailoring based on multiple or intersecting characteristics of the target population is related to greater effectiveness of the message (Uskul & Oyserman, 2010). This suggests the importance of addressing intersectionality – the dynamic interplay between the various identities held by an individual – in the tailoring of health messaging (Crenshaw, 1991). To such areas of intersectionality important for adolescents include culture and age.

#### Cultural Tailoring

There is robust literature suggesting the benefits of culturally tailored health messages in influencing viewers' healthy behaviors (Uskul & Oyserman, 2010). Culturally tailored health messages have been found to be effective in adolescent populations (Hall et al., 2016). These culturally tailored interventions rely on communication styles used in the target audience (e.g., naming intervention skills after popular hip-hop songs), center cultural values (e.g., the importance of family and gender-role expectations), and bolster existing culturally specific protective factors (e.g., nutritional value of heritage cultural foods) (Simenec et al., 2023; Anderson et al., 2018). In doing so, culturally tailored interventions support acceptability and retention (Bernal et al., 1995).

#### Developmental Tailoring

Although there is established evidence suggesting the utility of tailoring health messaging to match the audiences’ worldview, most literature focuses on adult populations without developmental considerations. However, age is suggested as a moderator in health communication and decision-making, and research suggests that older and younger adolescents differ in the ways they make decisions about their health (Koenig et al., 2020). These developmental differences within adolescence may be related to pubertal onset and interact with other contextual and environmental considerations (Koenig et al., 2020). Further, adolescent health autonomy gradually and linearly increases from age 9 to 20 (Wray-Lake et al., 2010). Significant biopsychosocial influences across the adolescent period may impact the ways that younger and older adolescents evaluate culturally adapted health (Wray-Lake et al., 2010). For example, in a study investigating acceptability of a culturally adapted, digital health intervention for Somali American adolescents, older adolescents rated the culturally adapted aspects of the intervention as more acceptable than younger adolescents (Simenec et al., 2024).

### Message Tailoring and AI

The use of AI to support preventative research has primarily included machine learning to support early diagnosis or identification of health risks (Bernert et al., 2020; Rowe & Lester, 2020). With the promising advancement of AI tools supporting prevention science and practice, little research has explored the potential of using these technologies to support the design and development of tailored intervention materials. This application of AI is especially rich for investigation to solve pragmatic problems related to health message tailoring that have been criticized in previous research including the high degree of financial capital and specialized personnel required to facilitate tailoring for multiple populations (Balci et al., 2022). Human-centered AI, in which humans interface with computer learning to complete complex tasks, can support the efficient development and design of tailored DTC advertisements to motivate adolescents to engage in online health programs (Ford et al., 2015). Design components such as script writing with culturally or developmentally tailored language, representation of characters using AI image generators, or voiceovers in various languages and dialects can be supported with available web-based AI tools (e.g., ChatGPT, CapCut, Google AI).

Cutting edge research suggests strong potential for leveraging AI technologies to design effective health messages. For example, participants in a recent study favored pro-vaccination messages developed using AI compared to human-generated messages created by the Centers for Disease Control and Prevention (Karinshak et al., 2023). The effectiveness of AI-produced health messaging has been replicated in various public health domains including adolescent smoking sensation and road safety for adult Arabic speakers (Hussain et al., 2025; Leung et al., 2025). As the availability and accessibility of human-centered AI technologies continues to expand, more research is necessary to determine how to leverage these tools to increase the effectiveness of health messages through tailoring.

### Elaboration Likelihood Model

Unsurprisingly, as with health decision-making, the effectiveness of DTC health messages differs by individual and contextual influences like developmental stage and culture. Research suggests that when a message matches an individual’s cultural context, they evaluate the message as more persuasive and *likeable* (Uskul & Oyserman, 2010). While the mechanisms involved in the persuasiveness of individualized health messaging remain understudied, the Elaboration Likelihood Model (ELM) presents one plausible explanation: the greater a message's *relevance* to the viewer, the more likely they are to evaluate and think critically about the content of the message (i.e., mental *elaboration*), which is related to more robust behavioral change (Petty & Cacioppo, 1986). Success of messages targeting health behavior change often measures *intentions* to engage in the behavior, which is highly predictive of actual performance of the behavior (Ajzen, 1985). For example, Oenema and colleagues (2005) used the ELM to investigate the effects of tailored nutrition education messages delivered in a digital intervention for adults in the Netherlands. They found that, related to the ELM, perceived personal relevance, individualization, and interestingness mediated the relationship between message tailoring and nutrition *intentions* (Oenema et al., 2005). However, support for the ELM in adolescent populations shows mixed findings for explaining the effectiveness of tailored health messages (Metzler et al., 2000; Te’eni-Harari et al., 2007). These differences may be related to variability in ELM measurement and study design in the few adolescent ELM studies. This study, therefore, uses AI to experimentally test the ELM prediction that message tailoring will enhance message relevance, elaboration, liking, and intentions to engage in a digital health intervention among Somali American adolescents.

### Current Study

The current study aims to investigate the effectiveness of DTC advertisements featuring an evidence-based, digital, food-focused media literacy intervention culturally adapted for Somali American adolescents called JUS Media? Global Classroom—Somali American (JMGC-SA; Simenec et al., 2024). The intervention promotes food-focused media literacy (the ability to think critically about unhealthy food advertisements) and nutrition knowledge using culturally and developmentally tailored health messaging. To our knowledge, this is the first study where publicly available AI tools supported the development of DTC advertisements. These AI-designed DTC ads were created in collaboration with the Somali American community.

Age stratified randomization was used to examine the four DTC ads varying in cultural and developmental tailoring. Based on prior literature we expected both cultural and developmental tailoring of DTC health ads would be associated with higher health message personal relevance (H1a), elaboration (H1b), liking of the message (H1c), and intentions to engage with JMGC-SA (H1d). Further, we predicted that there will be an interaction between tailoring factors such that DTC health ads that are both culturally tailored *and* developmentally tailored would account for a significant amount of variability in these four outcomes (H1e). Qualitative information adolescents’ qualitative opinions about the design components of the AI-developed health ads can be found in Supplement E.

## Method

### Participants

In total, 230 Somali American adolescents were recruited in a metropolitan area in Minnesota (M_age_ = 14.9; 67.5% female; 30.4% 1 st generation), home to the largest proportion of Somali American migrants in the United States. The data reported here represent the quantitative second phase of a sequential exploratory study; relevant results from the qualitative first phase are included in online Supplement A and full details are in Simenec et al., 2025. Inclusion criteria were ages 11 to 19 years, one parent born in Somalia, and comfort reading and writing in English.

### Procedure

Three research assistants from the Somali American community served as cultural brokers for participant recruitment. Recruitment occurred at a local charter school serving predominantly Somali American youth, at Somali American cultural events (e.g., health fair, community dinner), utilizing a participant pool from prior studies in this community, and snowball sampling. Aside from snowball participants who completed an eligibility screener, parents were contacted by a Somali American cultural broker to confirm eligibility, explain the study, and complete the parental consent process digitally with support of the cultural broker. Consented adolescents were emailed the link to the online survey that contained an embedded video advertisement. All participants assented before accessing study materials.

Data were collected from an online survey using Qualtrics. Participants first completed demographic, food and media knowledge, and cultural identity survey questions. Next, using stratified randomization by age (younger adolescents 11–14 years old/older adolescents 15–19 years old) participants viewed either a Somali American culturally tailored DTC health ad or a DTC health ad resembling health messages they typically see online. For participants receiving the Somali American culturally tailored ad, half viewed an ad developmentally tailored to match their age group. The same strategy was used to randomize participants to the typical ad, which also had younger and older versions of the ad. Each participant saw only one ad. After viewing the ad, participants then completed survey questions in response to the video ad they viewed.

### Advertisement Development

#### Tailoring Health Advertisements

**Fig. 1:**
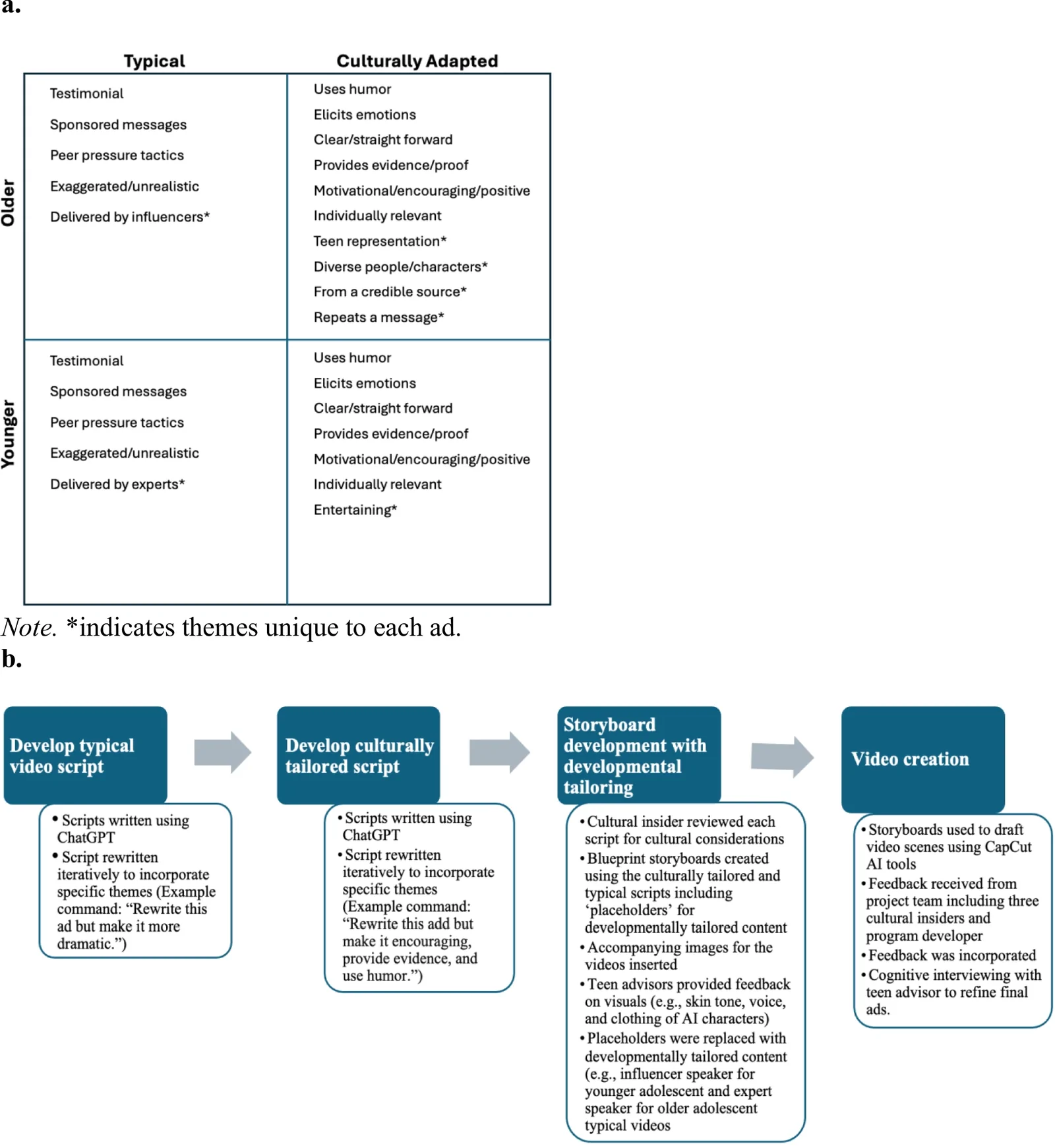
Culturally and developmentally tailored Ad design themes and Ad development process. *Note.* *indicates themes unique to each ad. Quadrants in Fig. 1a represent the themes potrayed within the design of each of the four tailored Ads to reflect cultural and developmental tailoring. Fig. 1b outlines the Ad development process.

Four DTC video ads tailored for Somali American adolescents were created for this study. All messages advertised the JMGC-SA intervention to Somali American adolescents and ended with encouragement to sign up for the program. Generating tailored health ads requires input from the target population to address specific factors impacting the target population’s health behaviors (Willis et al., 2022). Input from two Somali American teen advisors helped to inform and refine the four 1-min DTC video ads. See Fig. 1a for themes used in the design of each of the four culturally and developmentally tailored ads.

#### Culturally Tailored vs Typical Advertisements

Two culturally tailored ads were designed based on qualitative data from focus groups with Somali American adolescents collected in a previous study (Simenec et al., 2025). The culturally tailored ad for younger adolescents used themes such as humor, elicits emotions, individual relevance, and entertainment while the culturally tailored ad for older adolescents used themes such as teen representation, diverse people/characters, and from a credible source. The two typical ads were designed based on qualitative themes arising from the same focus groups as the culturally tailored ads and those data can be found in Online Supplement A. Specifically, instances of Somali American adolescents discussing types of digital health messages they typically see were thematically analyzed to inform the typical ads. These themes were used to guide the design of the ads for younger and older adolescents.

#### AI-Assisted Video Design

The DTC video ads were developed in four steps (Fig. 1b). The study used publicly available tools (e.g., Chat GPT; CapCut) to design and create all ads. This was done to identify a cost-effective, low labor process for creating tailored digital health ads. A blueprint storyboarding strategy was used to increase the efficiency of this digital design process while facilitating standardization within and between tailored versions of the ads (Simenec et al., 2023). Blueprint storyboarding creates a detailed plan to develop the intended media product and subsequent tailoring.

First, a script was drafted for the typical ad blueprint written using ChatGPT. The AI command used to write the first draft of the script focused on capturing the basic intent of the ad (i.e., “Write a 1-min script for a video marketing a media literacy intervention for teens.”). Then, commands were used to iteratively include overlapping themes between younger and older teens (e.g., exaggerated/unrealistic language, use of testimonials). This process was then repeated for the culturally tailored version of the video: commands were used to iteratively include overlapping themes between younger and older teens (e.g., provided evidence/proof for the claims made and used motivational language).

The remaining steps were completed in collaboration with a team of five Somali American cultural insiders including a hired research assistant, two undergraduate students supporting the project for course credit, and two teenagers were longstanding youth advisors in the lab. Scripts were reviewed by one cultural insider who provided feedback on cultural considerations. Two blueprint storyboards were then developed: one using the culturally tailored script and one using the typical script. Placeholders were inserted into each storyboard to indicate where developmentally tailored content was needed (e.g., influencer for younger adolescents and expert for older adolescents). Images were then inserted into both storyboards. At this stage, the two Somali American teen advisors supported selection of culturally resonate visual content including appearance of AI characters (e.g., skin tone, voice, and clothing). Placeholders were then replaced with developmentally tailored content. Finally, the ads were drafted (by the first author), and reviewed by three cultural insiders (including the second author) and the JMGC-SA program developer (anchor author). Finally, cognitive interviewing of one teen advisor was performed for all four ad drafts to ensure all the desired themes were appropriately conveyed. This step-by-step process by which Chat GPT was used to write the script for the typical videos using themes from the focus group and CapCut AI tools were used to develop the videos is detailed in Supplement B including AI commands used to draft the ad scripts.

After ad creation a pilot test with 42 individuals was conducted to ensure that the intended themes were adequately portrayed in the design of each of the four ads. More information about the pilot test can be found in Supplement C.

### Measures

#### Cultural Identity

Cultural orientation to three cultural identities was measured using the Identity subscale from the Language, Identity, and Behavioral Acculturation Scale (Birman et al., 2010). The scale measures tridimensional identity acculturation to Somali heritage culture, African American culture, and Mainstream White American using different items for each subscale (e.g., “I think of myself as being Somali/African American/White American”). Items were responded to on a 4-point likert scale from “Not at all” to “Very much.” A mean score was computed for each cultural orientation (M_Somali_ = 3.84, SD = 0.37; M_AfricanAmericani_ = 3.07, SD = 1.01; M_MainstreamWhiteAmericani_ = 1.30, SD = 0.72) and each subscale had adequate to excellent reliability (α = 0.81—0.96).

#### Personal Relevance

Acceptability of the advertisement was measured using a 16-item acceptability scale assessing the personal relevance of the ad (e.g., “The people in the videos *looked* like me or someone in my community.”) informed by the Ecological Validity Model (Bernal et al., 1995). Participants respond on a 5-point likert scale from “Not at all” to “Very much”. This strategy for measuring “fit” or personal relevance of culturally tailored health content has been used successfully in other research with Somali American adolescents (Simenec et al., 2024). The scale had strong reliability in the current sample (α = 0.91). A mean score was computed (M = 3.64, SD = 0.76).

#### Health Message Liking

Ad liking was measured using a 3-item self-report measure of adolescents’ liking of the ad using three semantic differential scales (i.e., “The video I just watched was [bad/good], [dislikeable/likable], [unfavorable/favorable]”). Participants respond on a 9-point likert scale using each of the three sematic differentials (Huhmann & Albinsson, 2012). The scale had strong reliability in the current sample (α = 0.90). A mean score was computed (M = 6.73, SD = 1.95).

#### Intentions to Engage in JMGC-SA

Intentions to engage in the online food-focused media literacy intervention, JMGC-SA, were measured using a 3-item self-report measure from the Theory of Planned Behavior Questionnaire (e.g., “I intend to use the online health program I saw in the video.”, “I will try to use the online health program I saw in the video.”, “I plan to use the online health program I saw in the video.”). Participants responded on a 7-point Likert scale from “strongly disagree” to “strongly agree”. These questions were adapted from a previous study evaluating intentions to seek help for mental health in Chinese adults (Mo & Mak, 2009). The Theory of Planned Behavior Questionnaire has been validated and used widely to study health behaviors in adolescent populations (McEachan et al., 2011). The scale had strong reliability in the current sample (α = 0.90). A mean score was computed (M = 4.7, SD = 1.48).

#### Elaboration

The degree to which participants elaborated on the ad was measured using a thought listing task. Participants were asked “Please write the thoughts you had as you watched the video below. Don’t worry about grammar or spelling.” Thought-listing measures are commonly used in communications and advertising research (Briñol et al., 2004; Shen & Seung, 2018). Responses were scored both quantitatively and qualitatively. First, responses were divided into distinct thought units and then the number of thought units was summed for a total elaboration score (M = 1.49, SD = 1.34; coding procedure followed described in Shen & Seung, 2018).

### Plan of Analyses

Based on the ad to which each participant was randomly assigned, each participant received a dummy-coded score for cultural tailoring and developmental tailoring. Separate hierarchical regression analyses were conducted regressing each DV on IVs (analytical sample n = 230). DVs included health message personal relevance (H1a), elaboration (H1b), liking (H1c), and intentions to engage in JMGC-SA (H1d). In each regression, sociodemographic variables significantly associated with any DVs or IVs were included in the first step as covariates (Model 1). In the second step, cultural tailoring and developmental tailoring were entered to assess for main effects (Model 2). In the third step, the cultural tailoring X developmental tailoring interaction term were included to assess effects of culturally tailored and age-matched messages (H1e; Model 3). This analytical method was selected to identify the unique variance accounted for each block of variables and to decrease issues of colinearity.

## Results

Multiple cultural identity variables were significantly correlated with outcome variables in the study (see Table 1). Specifically, Somali cultural identity was significantly and positively correlated with advertisement liking (meaning liking of JMGC-SA ads regardless of ad tailoring) and mainstream White American cultural identity was significantly and positively associated with intentions to engage with JMGC-SA. Therefore, all cultural identity variables in addition to gender were included as covariates in the hierarchical regression models.

**Table 1:** Correlations between study variables

Variables	1	2	3	4	5	6	7	8	9	10
1. Elaboration Likelihood Model	-									
2. Acceptability	.084	-								
3. Advertisement liking	.080	.559**	-							
4. Intentions	-.008	.492**	.521**	-						
5. Cultural Identity Somali	.036	.096	.206**	.119	-					
6. Cultural Identity African American	-.101	.142*	.111	.157*	.202**	-				
7. Cultural Identity Mainstream White	-.041	.060	-.037	.147*	-.186**	.113	-			
8. Country of Birth	.251	.062	.040	.021	.069	-.106	.039	-		
9. Age	.094	-.099	-.030	-.044	-.026	.095	-.111	.178	-	
10. Total Device Ownership	.096	-.028	-.022	-.096	-.048	-.137*	.078	.089	.051	-

p values reflect robust p values calculated from robust estimators of standard errors

^*^p <.05. **p <.01. ***p <.001

### Personal Relevance

In partial support of H1a, when regressing personal relevance on main effects in model two, cultural tailoring was significantly associated with higher reported personal relevance of ads viewed (β = 0.23, *p* =.03). Developmental tailoring, cultural X developmental tailoring, and covariates were non-significant, contrary to H1a and H1e. Model 2 performed best (*Adj R*^*2*^ = 0.34; Table 2).

**Table 2:** Hierarchical regression results for personal relevance

Predictor	Model 1 β	Model 2 β	Model 3 β
Covariates			
Gender	.10	.12	.13
Cultural Identity Somali	.19	.18	.19
Cultural Identity African American	.09	.09	.09
Cultural Identity White American	.07	.07	.07
Cultural Tailoring	—	.23*	.12
Developmental Tailoring	—	-.06	-.16
Cultural Tailoring × Developmental Tailoring	—	—	.22
Intercept	2.51	2.45	2.48
Model Summary			
Explained variance [*R*^*2*^*/Adj R*^*2*^ (%)]	36/19	59/34	64/34

p values reflect robust p values calculated from robust estimators of standard errors * *p* <.05. ** *p* <.01. *** *p* <.001

### Health Message Liking

In partial support of H1b, when regressing liking on main effects and interactions in model three, cultural tailoring (β =.77, *p* =.04), and Somali identity (.94,.01) were significantly and positively associated with ad liking. Developmental tailoring, cultural X developmental tailoring, and other covariates were non-significant, contrary to H1b and H1e. Model 2 performed (*Adj R*^*2*^ = 0.57; Table 3).

**Table 3:** Hierarchical regression results for liking

Predictor	Model 1 β	Model 2 β	Model 3 β
Covariates			
Gender	.25	.32	.31
Cultural Identity Somali	1.01**	.95*	.94*
Cultural Identity African American	.14	.14	.14
Cultural Identity White American	-.03	-.03	-.04
Cultural Tailoring	—	.69**	.77*
Developmental Tailoring	—	.02	.10
Cultural Tailoring × Developmental Tailoring	—	—	-.15
Intercept	2.38	2.26	2.24
Model Summary			
Explained variance [R2/Adj R2 (%)]	52/35	82/57	83/53

Note. p values reflect robust p values calculated from robust estimators of standard errors

^*^ *p* <.05. ** *p* <.01. *** *p* <.001

### Intentions to Engage in JMGC-SA

In partial support of H1c, when regressing intentions to engage in JMGC-SA on main effects in model two, cultural tailoring was associated with higher intentions to engage in JMGC-SA (β = 0.38, *p* =.05). When regressing intentions to engage in JMGC-SA on all main effects and interactions in all models, higher reported identification with mainstream White American culture was related to higher intentions to engage with the JMGC-SA program (β = 0.32, *p* =.02). Developmental tailoring, cultural X developmental tailoring, and other covariates were not significant, contrary to H1c and H1e. Model 2 performed best (*Adj R*^*2*^ 0.51; Table 4).

**Table 4:** Hierarchical regression results for intentions

Predictor	Model 1 β	Model 2 β	Model 3 β
Covariates			
Gender	-.19	-.15	-.14
Cultural Identity Somali	.52	.46	.47
Cultural Identity African American	.15	.15	.15
Cultural Identity White American	.32*	.32*	.32*
Cultural Tailoring	—	.38*	.23
Developmental Tailoring	—	.11	-.04
Cultural Tailoring × Developmental Tailoring	—	—	.30
Intercept	1.89	1.85	1.89
Model Summary			
Explained variance [R2/Adj R2 (%)]	59/42	76/51	79/49

Note. p values reflect robust p values calculated from robust estimators of standard errors

^*^ *p* <.05. ** *p* <.01. *** *p* <.001

To explore the cultural tailoring effect further, we then ran a post hoc regression analysis using standard covariates to explore whether Somali identity moderated the effects of cultural tailoring on intentions to engage with JMGC-SA. Somali identity strengthened the effect of cultural adaptation on intentions to engage with JMGC-SA (β = 1.59, *p* =.01; Figure in Supplement D).

### Elaboration

Contrary to H1d and H1e, there were no significant main effects nor interaction effects for elaboration after viewing the ad. None of these models explained a meaningful amount of variance (Table 5).

**Table 5:** Hierarchical regression results for elaboration likelihood model

Predictor	Model 1 β	Model 2 β	Model 3 β
Covariates			
Gender	-.16	-.15	-.14
Cultural Identity Somali	.20	.18	.18
Cultural Identity African American	-.15	-.15	-.15
Cultural Identity White American	-.04	-.04	-.03
Cultural Tailoring	—	.12	.05
Developmental Tailoring	—	.03	-.04
Cultural Tailoring × Developmental Tailoring	—	—	.13
Intercept	1.32	1.31	1.33
Model Summary			
Explained variance [*R*^*2*^*/Adj R*^*2*^ (%)]	18/0.1	20/−0.7	21/−1.1

Note. p values reflect robust p values calculated from robust estimators of standard errors

^*^ *p* <.05. ** *p* <.01. *** *p* <.001

## Discussion

This study tested culturally and developmentally tailored ads created with the support of publicly available AI tools designed to persuade Somali American adolescents to use a digital health intervention (JMGC-SA). Results suggest that cultural adaptation of the digital health ads significantly improved participants' reported personal relevance, liking, and intentions to engage in the digital intervention partially supporting hypotheses H1a, H1c, and H1d. However, developmental adaptation did not significantly impact any of the primary study outcomes nor interact with cultural adaptation contrary to H1e. Findings did not support the Elaboration Likelihood Model (ELM) in this sample of adolescents contrary to hypothesis H1b. Findings also revealed that Somali cultural identity predicted higher liking of the ad and amplified the impact of cultural tailoring on intentions to engage with the JMGC-SA, while stronger mainstream White American cultural identity was related to greater intentions to engage with the program.

### Cultural Adaptation

Cultural tailoring of the digital health ads significantly increased three of the four primary indicators of program effectiveness for Somali American adolescents – personal relevance, liking, and intentions to engage in a digital intervention. This finding supports a meta-analysis of the benefits of cultural adaptation of health messages in adults and adolescent populations from diverse cultural groups (Hall et al., 2016). The current study builds on this previous research by supporting the effectiveness of cultural adaptation in direct comparison to typical, non-adapted messaging using an experimental design given that many studies supporting cultural adaptation of health ads in adolescent populations do not include a comparison group (Hall et al., 2016).

On the other hand, cultural adaptation of the digital health message was not predictive of evaluation similar to other research on the ELM in adolescence, suggesting a new model is needed to understand the effects of tailored health messaging in adolescent populations (Te’eni-Harari et al., 2007). Research calling for extensions of the ELM that accounts for children and youth cognitive and social capacities required for systematic information processing renders traditional ELM assumptions less relevant. A more developmentally-sensitive model of cognitive engagement that accounts for quality of thoughts in response to a digital health message rather than quantity as elaboration is typically measured may help to explain these findings – such as the young people’s processing of commercial media content models (PCMC) – may therefore better explain how adolescents engaged with tailored digital health messages in evolving media contexts (Buijzen et al., 2010).

Findings from this study challenge the notion that cultural adaptation of digital health messages is not worth the invested time and financial resources (Balci et al., 2022). AI tools in partnership with cultural insiders supported efficient design of culturally tailored ads with few resources: all developed in two months by graduate student researcher with a moderate level of video design skills, two undergraduate cultural insiders supporting the project for course credit, and two teen advisors who were compensated $50 each for their expertise (about 2 h).

The current study found that regardless of ad tailoring, Somali cultural identification was associated with higher ad liking and mainstream White American cultural identification was associated with higher intentions to use the digital health program. These findings align with previous research suggesting that the effectiveness of digital health messages differs by contextual influences like cultural identity. For example, Danish and Hong Kong adolescents scoring higher in collectivism show a higher level of liking and reported effectiveness of five print advertisements encouraging less soft drink consumption than did adolescents who scored higher on individualism regardless of the ad content (Chan et al., 2010). Further, findings support previous research suggesting that racial identity beliefs of Black young adults impact the desirability of engaging with digital health resources (Willis & Neblett, 2023). Importantly, the JMGC-SA program targets consumption of mainstream White American media as a risk factor for unhealthy eating while bolstering Somali heritage culture as a buffer or protective factor. Notably, two cultural orientations – Somali and mainstream White American, but not African American – impacted the effects of the health ads, which was supported by measuring each cultural orientation separately from a tridimensional acculturation perspective (Ferguson et al., 2021). Further, culturally adapted ads were more effective for participants who identified closely with their Somali culture suggesting that cultural tailoring may be especially important for supporting the health of individuals who are connected with their heritage culture.

### Developmental Adaptation

Developmental adaptation of the ads did not significantly predict any of the tested outcome variables in this study (personal relevance, liking, and intentions to engage in the digital intervention). The lack of support for this hypothesis may be because our 11–19-year-old sample were all in the final stage of consumer skill development, according to consumer socialization theory, despite this being a broad chronological age range (John, 1999). In the reflective stage, children have knowledge about marketing concepts such as branding and are able to reflect and reason about their role as consumers as they make adaptive choices (John, 1999). Additionally, this generation of adolescents are digital natives and likely have extensive experiences with digital ads with abundant opportunities to develop consumer skills and preferences—with more recent studies suggesting children are far beyond the perceptual stage of development by kindergarten (Atkinson et al., 2015). This means that even if we had sampled younger youth, they may also have already advanced into the same reflective stage. Therefore, all participants in this study are likely to have achieved similar consumer development and experiential learning that limited assessment of developmental differences in the design of the tested ads. An alternative explanation may be that the impact of cultural adaptation of the ads (e.g., individual relevance; motivational/positive/encouraging) was especially impactful and masked the effects of more surface level developmental adaptation of the ads (e.g., entertaining; repeats a message). This finding suggests that developmental tailoring is not needed to effectively reach and engage Somali American adolescents with culturally tailored digital health prevention resources.

### Implications

Artificial intelligence is increasingly prominent in the field of health promotion and intervention, utilizing machine-based systems to support adaptation efforts by reducing resource barriers and amplifying the adaptability provided by health ads to *efficiently* tailor for multiple cultural communities (Simenec et al., 2023). This work extends on evidence suggesting the effectiveness of AI-developed health promotion messages by demonstrating the value of culturally and developmentally tailored message content and design to ensure both effectiveness and reach. These findings emphasize that by leveraging the strengths and perspectives of Somali American adolescents, researchers and practitioners can ensure that health prevention efforts are not only culturally relevant but also foster a sense of ownership and empowerment. It is critical for future research and practice to continue identifying strategies to increase the cultural responsiveness of digital health ads to avoid replicating existing disparities in dominant healthcare systems (Willis et al., 2022). Policy, research, and practice should ensure that tailored health ads for Black adolescents address social determinants of health and are co-created with the community members to ensure relevance and effectiveness (Joseph & Fleary, 2025).

Ethical limitations of AI should be considered when using AI as a tool to support cultural adaptation of DTC health ads. Machine learning that is used to guide AI output is based on the inputs these programs are trained on, which can include systematically racist information (Rowe & Lester, 2020). This same racist information could lead to ad designs that reinforce current systemic injustices in the healthcare system. Ethical guidelines recommend that digitally-based research with Black migrant adolescents and families require community based participatory research strategies to ensure cultural responsiveness and avoid harmful experiences with health institutions that have historically and presently mistreated these communities (Hodges et al., 2024). Therefore, it is unethical to use AI alone without input from cultural experts and community members to develop culturally tailored health products. Experts in prevention and intervention capitalizing on the utility of AI tools should always do so in collaboration with input from members of the community. Driven by these principles, the current study engaged with Somali American teen advisors as well as cultural brokers to ensure accurate and culturally responsive ad tailoring. Evidencing that co-production of health messages in collaboration with end-users is in line with the growing body of literature on leveraging health promotion using AI tools (see Rice & Young, 2025 and Smith et al., 2024).

### Limitations and Future Directions

The current study contributes to the body of research on the use of AI in developing culturally tailored DTC ads to disseminate health prevention materials. However, interpretation of study findings should account for limitations. The study measured participant-reported intentions to engage with the digital health program after viewing the ad rather than intervention engagement or other health behaviors. It is unknown whether participants followed through with these intentions to engage with the health program. Future research should include measures of health behaviors after viewing tailored health messages. The study design employed an experimental study design by showing participants a DTC ad within a digital survey. This study design allowed for systematic testing of ad performance that would not be possible if ads were delivered to adolescents directly through digital media platforms (e.g., social media), which may impact ad effectiveness. Future research should extend the external validity of findings to examine the effectiveness of these ads when delivered through digital media.

## Conclusion

As the digital age continues to boom, prevention efforts to deliver digital health resources directly to Black and migrant youth has increased across a breadth of targeted health domains (Joseph & Fleary, 2025; Simenec et al., 2024). Digital tools are not only aligned with the way today's adolescents access information, but they also provide a platform for overcoming barriers such as geographic isolation or language limitations. Designing digital health ads with support of accessible AI tools and in collaboration with adolescent cultural insiders increases the potential to expand the development of culturally tailored ads. Findings from this study reinforce the effectiveness of culturally tailored messaging compared to non-tailored ads, and the potential of AI tools in creating scalable digital health messaging for multicultural migrant and refugee youth. By leveraging AI tools, prevention programs can reach adolescents where they are—online—and deliver culturally relevant health content in formats that resonate with them.

## Supplementary Information

Below is the link to the electronic supplementary material.

Supplementary file1 (DOCX 58 KB)

## Data Availability

For transparency, requests for deidentified quantitative data made to the senior author (PI) by fellow researchers engaged in similar research may be considered if they do not compromise participant confidentiality or beneficence.
