# Supplementary material for: Disseminating Health Prevention Programs Using AI-Crafted, Culturally Tailored Ads for Somali American Adolescents
Source: Prev Sci. 2026 Mar 18;27(7):1111–24. doi: 10.1007/s11121-026-01891-6 (PMC13550089; doi:10.1007/s11121-026-01891-6)
Supplement: Supplementary file 1 [file 11121_2026_1891_MOESM1_ESM.docx]

**Disseminating Health Prevention Programs Using AI-Crafted, Culturally Tailored Ads for Somali American Adolescents**

*Prevention Science*

*Tori Simenec, Salma Ibrahim, Gail Ferguson*

University of Minnesota

Corresponding author: Tori Simenec; [sime0060@umn.edu](mailto:sime0060@umn.edu)

**Supplement A**

***Typical Ad Themes***

**Participants**

Inclusion criteria for the qualitative focus groups were: ages 11 to 17 years were one parent born in Somalia, comfort speaking in English. Twenty first- or second-generation immigrant Somali American adolescents ages 12 to 17 (M_age_ = 14.35 years; 55% female) participated in focus groups.

**Planned Analysis**

Parallel to Simenec et al (in press) that generated themes for the culturally tailored ads, for the two typical ads, thematic analysis of focus group data (Braun & Clark, 2006) was conducted to identify what design components were salient to Somali American adolescents. A consensual coding approach was used: coding was completed by one cultural insider (Somali American - second author) and one cultural outsider (White American, first author). First, these coders familiarized themselves with the entire corpus of data, then they employed inductive coding strategies to assign codes independently, after which they met to establish consensus and resolve discrepancies through discussion. Next, codes were organized into broad themes.

**Results**

Thematic analyses of focus group data revealed some differences between younger and older adolescents in the themes they perceived in typical health messages they view online, which were used to design the two typical ads. The typical ad for younger adolescents included themes such as “delivered by influencers” and “peer pressure tactics” whereas the typical ad for older adolescents included themes such as “delivered by experts” and “exaggerated/unrealistic”. See Figure 1 for the complete list of themes included in each ad.

**Supplement B**

**Step-by-step Process to Create Tailored Video Ads**

Step 1: Chat GPT was used to write the script for the typical videos using themes from the focus group.

a. AI Commands:
   i. “Write a 1 minute script for a video marketing a media literacy intervention for teens”
      A. Note: first script already included testimonials and used language similar to a sponsored message
   ii. “Write it again, but targeting teens and for a video-based intervention”
   iii. “Write it again but make the focus on media literacy for junk food ads”
   iv. “Write it again but have the narrator be a nutrition influencer”
   v. “Combine the last two scripts to focus on media literacy for junk food ads with an influencer narrator”
   vi. “Rewrite this ad but make it more dramatic”
   vii. “Rewrite this ad but make the claims more unrealistic”
   viii. “Rewrite it without mentions of space. Make the goals for nutrition unrealistic”
   ix. “Rewrite this ad but make the claims of the program more extreme”
   x. “Rewrite this ad, but include an element of peer pressure”

Step 2: Chat GPT was used to write the script for the culturally tailored videos using themes from the focus group.

a. AI Commands:
   a. “Write a script for a 1-minute ad for an online food-focused media literacy intervention for teens that is encouraging, provides evidence, and uses humor”
      i. Note: this script only needed minor tailoring after the first draft.
   b. “Rewrite but focus on junk food ads”

Step 3: Storyboard development

b. A cultural insider reviewed each script to tailor for developmental and cultural content (e.g., using generationally relevant language).
c. The final scripts for the culturally tailored and typical versions of the message were developed into blueprint storyboards.
d. Accompanying images for the videos were inserted.
e. b. Each blueprint was then developmentally tailored.
   a. The cultural insider and project lead worked together to identify appropriate scenes to include guided by differing themes between age groups.
   b. The project lead went through iterative revisions with cultural insider on all versions of the storyboards before drafting the videos.

Step 4: Videos were drafted using CapCut

a. Storyboards used to draft video scenes
b. Teen advisors provided feedback to select appropriate visual content including appearance of AI characters (e.g., skin tone, voice, and clothing considerations)
c. Once drafted, received feedback from project team including three cultural insiders
d. Received feedback from program developer
e. Received feedback from teen advisor using cognitive interviewing to confirm themes were communicated appropriately
f. Revised videos to include emphasis on expert credentials, use of popular gifs, change in AI voice/dialect, improved visuals/transitions

**Supplement C**

**Tailored Ad Pilot Test**

A pilot test of the four tailored ads used in this study was completed using a convenience sample of 42 individuals. Each individual was randomized to view one of the four tailored videos and then rate the degree of fit to each of the intended themes using a 5-point likert scale from “Strongly Disagree” to “Strongly Agree”. For example, “The video message I just viewed included a testimonial”. Data were analyzed using a one-way ANOVA with four factors (for each of the four videos) to examine if there were significant differences in endorsement of the expected themes across videos. Descriptive statistics were then used to assess if the direction of differences in means was in the indented or unintended direction of the manipulation.

Results of this pilot test indicated that three of the four ads were evaluated as having strong fit to expected themes at a statistically significant level. Ratings for the themes of clarity, *F*(3, 40) = 3.65, *p* = 0.02, and motivational, *F*(3,40) = 3.14, *p* = .04, for the culturally tailored younger teen version were significantly lower than expected in the culturally tailored younger version of the ad given these themes were unique to this video. Edits were made to this video to account for these discrepancies (i.e., lowering music and slowing pace for clarity and adding an encouraging line at the end of the video). The culturally tailored younger teen version did not show a difference in the only unique theme – entertaining, F(3, 40) = 0.48, *p* = 0.7. Because this was the only unique criterion for this video, it was edited to increase the entertainment value of the video (e.g., sound effects, pacing, animation). Finally, the theme of diversity did not suggest a difference between the younger and older culturally adapted videos F(3, 40) = 1.43, *p* = 0.25. Given the study focus of cultural adaptation, the culturally tailored older teen version was tailored to increase the presence of diverse people.

**Supplement D**

**Figure 1.** Interaction between Cultural Tailoring and Somali Cultural Identity in Predicting Intentions

***Note.*** Figure represents the moderation without the inclusion of covariates showing that at high levels of Somali cultural identity (+1 SD), the simple slope was significant and positive (β = 0.99, *p* = 0.001) whereas it was non-significant and negative at low levels (-1 SD, β = -0.16, *p* = 0.578)

**Supplement E**

**Participant Opinions of the AI-Developed Ads**

Adolescents’ qualitative opinions about the design components of the AI-developed health ads were thematically analyzed. We expected that adolescents’ elaborated qualitative responses would reflect liking of the culturally tailored aspects of the ads. Evaluation of the use of AI in the ads was an exploratory question; therefore, there were no a priori expectations regarding adolescents’ opinions regarding that specific design component.

**Qualitative Analyses**

Thematic analysis was conducted on the content of adolescents’ responses to the elaboration question in the online survey across all four ads (see measure) (Braun & Clark, 2006). All responses containing thought units relevant to design components of the ads (analytic sample n = 208) were coded; distinct thought units, rather than entire responses, were the units of coding (i.e,, one adolescent response may contain multiple distinct thought units resulting in multiple codes). Coding was conducted by two coders (both Somali American - second author and research assistant) who independently employed inductive coding strategies. Afterwards, these two cultural insider coders met with a cultural outsider coder (White American - first author) for a consensual coding discussion where they resolved discrepancies, established consensus, and grouped similar codes into overarching themes then superordinate domains.

**Qualitative Results**

***Ad Design Component Themes***

Thematic analyses of participants’ responses to the elaboration measure identified 24 codes (italicized in the text), which were grouped into 7 themes, which were further collated into two domains called “Likes” versus “Dislikes” because adolescents expressed a range of positive and negative reactions to the AI-developed video ads. The Likes domain included three themes Positive Engagement, Message & Content Approval, and Design Element Approval*)* and the Dislikes domain contained four themes (Negative Engagement, Barriers to Message Approval, Design Element Disapproval, and AI-Related Disengagement).

***Likes***

***Positive Engagement.*** Within the theme of Positive Engagement, adolescents’ responses showed that they were positively engaging with the ad, often expressing immediate, *surface-level* impressions without deeper reflection or insights on its message or content. They described it as “cool,” “very nice,” and “really interesting.” Some showed an *emotional response*, including “it’s a nice, calm video” and feeling “speechless, astonished.” emotional reactions were often directed toward the facts presented in the ad, such as “I was shocked about the amount of ads we are exposed to everyday,” including emotional reactions to culturally tailored aspects of ads such as recognizing how the ad was tailored to them: “it was somewhat moving as I was part of the audience that the video was targeting.” Relatedly, responses highlighted that adolescents appreciated the ad for promoting *learning and education*. Many noted they “learned things [they] didn’t know before,” especially regarding healthy eating habits and media literacy. Furthermore, the ad sustained general *interest*, with numerous adolescents noting it was “very interesting” and that it was “very intriguing and [they] didn’t know it was that deep.”

***Message and Content Approval.*** Adolescents showed general *agreement* with the ad messages, such as its focus on supporting Black children with health-related content. They also found the ads credible. One adolescent remarked on the *credibility*, referring to the JMGC-SA being advertised in the ad, “They had 2 sources and so a part of me thinks the information they gave me was credible.” The ad’s *advertising persuasiveness* was also noted, with participants appreciating “the way the information was portrayed,” and that it was “advertised well.” Furthermore, the *practical impact and usefulness* of the ad resonated with them, as it provided new knowledge and takeaways. One adolescent emphasized the importance of the message, stating, “It’s important that it focuses on educating youth and parents who get exposed to US advertisements on unhealthy diets, to instead [make] healthier choices.”

***Design Element Approval*.** The codes with this theme reflected the production quality and key features of the advertisement that adolescents enjoyed, some of which related directly to the cultural tailoring (Figure 1). First, the ad’s *brevity* was appreciated, with one adolescent calling it a “good 1-minute video”. *Humor* also was noted as well-received, with one adolescent saying “I loved it *laughing emoji* so funny wlh” (“wallahi,” shortened as *wlh*, is a Somali American colloquial expression that means "I swear to God" used to add emphasis or to promise that something is true) and another saying “It was a funny video”. Additionally, the importance of the *persons* represented in the ad, such as Somali individuals was positively regarded: “I had a strong connection with the Somali people in the ad as they were people I’m used to seeing and feel safe around.” Finally, the ad *editing* was praised, with comments such as “amazing editing,” and “...editing skills were so good”, further demonstrating positive perception of the ad’s design and presentation.

***Dislikes***

***Negative Engagement.*** For the theme of negative engagement, the reactions ranged from general dissatisfaction to complete disengagement. Some adolescents experience a *general negative reaction*, responding that they thought the ad was “a bit weird” or “really corny and cringey,” or generally communicating that the message felt off-putting. Others noted *active disengagement* while viewing the advertisement, explicitly rejecting its content or even expressing they would take steps to remove it from their digital space: —“If I was to see this on my [page], I would report and scroll.” *Passive disengagement* was marked by indifference or disinterest, where adolescents neither strongly rejected nor engaged with the content, as two adolescents noted: “I didn’t really think nothing of it” and “I didn’t care about it.”

***Barriers to Message Approval****.* Adolescents identified aspects of the ads that hindered their acceptance of a message. *Skepticism* was noted as some adolescents expressed that the ads lacked credibility that led to distrust. For example, one adolescent wrote that “The overall quality and voicing of the ad didn't seem entirely real and made it seem untrustworthy as a believable informative video.” Similarly, participants pointed out areas of *content discrepancy*, where contradictions or missing information were evident. One participant shared, “it just seemed contradictory that they mentioned how all those food companies use influencers and celebs, and then use it for their own ad.” Finally, *engagement barriers* were detected regarding obstacles hindering the effective reception of the message, such as confusion or lack of clarity in the presentation: “It was an interesting concept but the delivery fell flat.”

***Design Element Disapproval****.* In this theme, adolescents expressed dissatisfaction with aspects of the design including language, characters in the ad, and production quality. *Language use* was critiqued as evident in this adolescent’s remark: “The slang was weird.” There were some related concerns about *persons*, or the individuals featured in the message: “like showing Travis Scott—it was weird.” Finally, *production quality* was another point of disapproval, as adolescents commented on the technical aspects of the message’s visual execution. One adolescent stated, “I thought the green screen was bad,” highlighting dissatisfaction with the visual effects or background editing. Another noted, “I think the ad is very good, but if it were more professionally done it would’ve been better received.”

***AI-Related Disengagement.*** AI-use was not widely recognized, but when it was, adolescents described it as contributing to a sense of detachment and disengagement. Some adolescents expressed *negative reactions toward AI-generated content*, describing it as unrelatable, off-putting, or difficult to engage with: “I did not like the AI at all. I hate the way it looked.” Additionally, the AI was cited as i*mpersonal and a mechanical experience* for some adolescents, who described the AI-generated content as lacking human presence and authenticity: "I was not really interested because I felt it was AI talking instead of a real person.” This sentiment was echoed by another adolescent who stated, “I really didn’t like the AI. And if you make it feel more human, people might actually sign up.” For some, the artificial nature of AI content made it less compelling, while others reported *disengagement triggered by AI* such as the AI voices in particular. For example, one adolescent noted, “Didn’t care about it. Their voice bothered me.” Across these responses, AI-generated elements shaped adolescents’ reactions, often leading to disinterest, disengagement, or outright rejection.

**Discussion**

The qualitative findings showed that participants enjoyed the ads for their engaging content, clear messaging, and appealing design. The culturally tailored design elements were generally liked versus disliked. Participants disliked aspects that felt unengaging, made the message harder to accept, or featured design elements they found ineffective. When they recognized AI, they tended to view it negatively, which further contributed to their disengagement. Taken together, these findings suggest that cultural adaptation of health ads developed with the aid of AI tools can effectively support dissemination of digital health resources, placing preventive resources into the hands of Black adolescents who may face barriers accessing traditional health services.

Qualitative findings also corroborate the role of cultural adaptation in shaping participants’ approval of health ads, particularly noted in their recognition and appreciation of design features that reflected their backgrounds and experiences through cultural tailoring. Participants especially valued the value of cultural tailoring of persons in the ads (Bernal et al., 1995; Simenec, Gillespie, et al., 2023), as reflected in the theme of Design Element Approval, with one noting a "strong connection with the Somali [characters] in the video." Additionally, within the themes of message & content approval and practical impact & usefulness, findings supported the role of cultural adaptation in enhancing message relevance, as one participant noted, “the video has a lot of informations that really no one tells you about ads, especially how it impacts us Somali-Americans," highlighting the value of content tailored to their community's unique experiences.

Although not many participants explicitly identified AI, when mentioned in qualitative responses, its presence was generally regarded negatively. To ensure the AI-generated content is responsive rather than merely targeted, it is crucial to prioritize authenticity by bridging AI design elements with human design elements. For instance, participants mentioned that voices sounded unnatural and mechanical, suggesting the need for voice actors rather than relying solely on AI-generated voices. While AI can be used in content creation, a careful balance may be needed to ensure it does not detract from the engagement or genuineness of the message.

To avoid priming, the elaboration response question prompt generating our qualitative data only inquired about general thoughts on the ads (versus specific thoughts on AI usage). Future qualitative research can examine more directly how many participants identify AI and their opinions on DTC messaging delivered by AI.

**References**

Bernal, G., Bonilla, J., & Bellido, C. (1995). Ecological validity and cultural sensitivity for outcome research: Issues for the cultural adaptation and development of psychosocial treatments with Hispanics. *Journal of Abnormal Child Psychology*, *23*(1), 67–82. https://doi.org/10.1007/BF01447045

Braun, V., & Clarke, V. (2006). Using thematic analysis in psychology. *Qualitative Research in Psychology*, 3(2), 77–101. <https://doi.org/10.1191/1478088706qp063oa>

Simenec, T. S., Ibrahim, S. A., & Ferguson, G. M., (in press). “You should always check… Make sure you’re not getting fed misinformation.” Somali American adolescents’ engagement and preference with digital health information. *Journal of Adolescent Research.*
